# Making Artificial Intelligence Work at Work: The Role of Human Resource Practices and Personal Attitudes in Fostering Meaningful Work with Artificial Intelligence

**DOI:** 10.3390/bs16020238

**Published:** 2026-02-08

**Authors:** Cataldo Giuliano Gemmano, Danila Molinaro, Diego Bellini, Silvia De Simone, Maria Luisa Giancaspro, Marina Mondo, Carmela Buono, Barbara Barbieri, Paola Spagnoli, Amelia Manuti

**Affiliations:** 1 Department of Educational Sciences, Psychology, Communication, University of Bari “Aldo Moro”, 70121 Bari, Italy; giuliano.gemmano@uniba.it (C.G.G.); amelia.manuti@uniba.it (A.M.); 2 Department of Human Sciences, LUMSA University, 00193 Rome, Italy; 3 Faculty of Social and Communication Sciences, Universitas Mercatorum, 00186 Rome, Italy; diego.bellini@unimercatorum.it (D.B.); carmela.buono@unimercatorum.it (C.B.); 4 Department of Pedagogy, Psychology, and Philosophy, University of Cagliari, 09123 Cagliari, Italy; desimone@unica.it (S.D.S.); mmondo@unica.it (M.M.); 5 Faculty of Human Sciences, Education and Sport, Pegasus Telematic University, 80143 Napoli, Italy; marialuisa.giancaspro@unipegaso.it; 6 Department of Political and Social Sciences, University of Cagliari, 09121 Cagliari, Italy; barbara.barbieri@unica.it; 7 Department of Psychology, University of Campania “Luigi Vanvitelli”, 81100 Caserta, Italy; paola.spagnoli@unicampania.it

**Keywords:** Artificial Intelligence, HR practices, work meaningfulness, attitudes toward AI, job performance

## Abstract

The rapid diffusion of Artificial Intelligence (AI) is transforming job characteristics, raising important questions about how to implement these technologies in organizations in ways that support employee well-being and performance. Drawing on the High-Involvement Management framework, this study examined employee-centered Artificial Intelligence implementation (ECAII) practices (defined as transparent communication, consultation, and training initiatives) as strategic levers to foster positive employee outcomes during Artificial Intelligence-driven transformations. Survey data were collected from 168 Italian white-collar employees who actively used Artificial Intelligence in their work. Structural equation modeling was employed to test direct and indirect relationships among employee-centered Artificial Intelligence implementation practices, work meaningfulness, job satisfaction, and job performance, as well as the moderating role of personal attitudes toward AI. Results showed that employee-centered Artificial Intelligence implementation practices had significant direct effects on both job satisfaction and performance, as well as indirect effects through work meaningfulness. Latent moderated mediation analyses further revealed that these indirect effects were stronger among employees with more positive attitudes toward Artificial Intelligence. Overall, the findings highlighted the importance of employee-centered strategies for enhancing meaningfulness and fostering positive outcomes during technological change. This study contributed to Human Resource Management (HRM) and meaningful work research by extending classic theoretical frameworks to Artificial Intelligence-enabled workplaces. Furthermore, from a practical perspective, our findings provided valuable guidance for organizations by highlighting the importance of transparent communication, employee involvement, and targeted training in reducing uncertainty and helping employees perceive their roles as relevant during the implementation of Artificial Intelligence.

## 1. Introduction

Artificial intelligence (AI) technologies are becoming embedded in organizations through various forms, including chatbots, recommendation systems, machine learning models, and generative AI. These technologies are not only supporting how work is performed but are also reshaping job characteristics, required competencies, and the way employees make sense of their work. Such transformations bring about opportunities in terms of efficiency gains, decision-making support, and innovative human–machine collaboration but simultaneously pose risks concerning resistance and loss of meaning at work (Smids et al., 2020; Valtonen et al., 2025). Unlike earlier automation and digitalization processes, AI is designed to simulate human intelligence through algorithms and extensive data, as well as performing complex tasks traditionally carried out by humans (Glikson & Woolley, 2020; Tahir et al., 2025; Zirar, 2023). As a result, it can alter the way individuals interpret their job roles and perceive the meaning of their work. In terms of epistemic authority (i.e., someone whose opinion is trustworthy because they have a better position of knowledge than others in a specific domain; Croce, 2018), AI puts algorithmic outputs ahead of human expertise, often making them appear more objective or reliable. This shift may lead employees to passively accept automated outputs instead of actively participating in a shared process of meaning-making (Shin, 2025). Furthermore, AI systems are opaque, which exacerbates these dynamics. Notably, deep learning models are commonly viewed as “black boxes” that generate outputs difficult to analyze or account for (Burrell, 2016; Lipton, 2018). Thus, employees may lose the ability to interpret work processes and experience displacement from decision-making autonomy and responsibilities.

In this context, human resource (HR) practices may play a pivotal role in the AI implementation process, as they can guide the human side of technological change. Specifically, HR practices can help employees understand, accept, and engage with AI-driven transformations in ways that foster well-being and productivity (Gama et al., 2022; Vishwakarma & Singh, 2023). In this vein, González-Romá et al. (2025) underlined the role of the organizational strategy during technological transitions by conceptualizing the construct of Employee-Centered Automation Implementation as a set of organizational practices aimed at actively involving employees in the process of automation adoption. Such an employee-centered strategy ensures that workers are informed, consulted, and trained throughout the process, thereby fostering job characteristics that are better aligned with employees’ needs and expectations, and ultimately contributing to higher levels of individual satisfaction. This perspective was consistent with a broader stream of research on participatory HR practices and employee involvement during organizational and technological change, which emphasized the importance of information sharing and skill development to support positive employee outcomes (Alqudah et al., 2022; Garmendia et al., 2021; Zhao et al., 2022). Similarly, studies on digital and AI-related transformations highlighted that transparent communication, employee voice, and training initiatives were critical for fostering acceptance, meaningfulness, and effective adaptation to new technologies (Bankins & Formosa, 2023; Gama et al., 2022; Jarrahi, 2018). In this sense, the contribution by González-Romá et al. (2025) can be seen as a recent integrative framework that systematized these principles within the specific context of automation implementation.

Building on this perspective, in the context of AI, Employee-Centered AI Implementation (ECAII) was defined as the extent to which organizations adopt practices that involve employees in the implementation of AI technologies through transparent communication, broad consultation, and adequate training. These employee involvement strategies may be particularly relevant not only for enhancing employees’ satisfaction and performance (Ahmad et al., 2014; Alqudah et al., 2022; Zhao et al., 2022), but also for fostering work meaningfulness in the context of AI adoption (Bankins & Formosa, 2023). Indeed, the introduction of AI represents both a challenge and an opportunity for employees, reshaping work content and skill requirements, while simultaneously opening new possibilities for improving employee experience and performance (Jarrahi, 2018; Jia & Hou, 2024). In this context, employee-centered strategies such as ECAII may play a crucial role in supporting employees during the transition, helping them to better understand and adapt to technological change, and ultimately fostering more positive attitudes, satisfaction, and performance outcomes (Bankins & Formosa, 2023; Jia & Hou, 2024).

Although HR literature recognized the importance of organizational practices in supporting employees’ experiences of organizational change (e.g., Boxall & Macky, 2009; Lawler, 1986), empirical research in the context of AI overlooked the collective dimension of employee involvement in organizational strategy. Most studies instead focused on the individual level, predominantly examining factors such as employees’ attitudes toward AI or AI technology acceptance (e.g., Almeida et al., 2025; Kaya et al., 2024). This lack of research limited our understanding of how organizations can foster positive employee outcomes in the context of AI-driven work. In particular, little was known about the psychological processes and mechanisms through which organizational practices could influence employees working with AI, leaving unanswered the question of whether and why ECAII may lead to beneficial outcomes such as job satisfaction and performance.

Moreover, an integrated perspective connecting organizational practices with individual attitudes was still lacking, preventing a full understanding of the mechanisms through which ECAII could promote positive outcomes while accounting for employees’ specific orientations toward AI. Specifically, little was known about the beneficiaries’ perspective, investigating if and to what extent they valued these practices as effective. Thus, it remained unclear whether employees’ personal attitudes toward AI may amplify or weaken the effects of ECAII, since the effectiveness of organizational practices could not be fully understood without accounting for their interaction with individual factors.

Addressing these gaps in our study was crucial from a theoretical and practical perspective. Theoretically, it advanced understanding of how organizational and individual factors jointly determined employees’ experiences of AI at work. An integrated organization–employee approach could clarify the mechanisms and boundary conditions through which ECAII translates into employee outcomes. This could be achieved both by identifying one of the mediating variables that could explain why ECAII fostered positive outcomes and by examining the interaction between the organizational and the individual perspective on AI. Such an effort could contribute to bridging authoritative organizational-level HR perspectives (e.g., Lawler, 1986; Nadler & Tushman, 1980) with psychological individual perspectives of AI acceptance (e.g., Knell & Rüther, 2024; Smids et al., 2020), ultimately enriching the theoretical foundations of research on AI in the workplace.

From a practical perspective, clarifying the role of organizational strategy and its interaction with employees’ attitudes toward AI was essential for organizations aiming to implement AI technologies successfully. Defining an implementation strategy that enhanced employees’ understanding and involvement, and identifying the individual conditions under which this strategy was most effective, could afford valuable guidance to managers and HR practitioners in fostering meaning, satisfaction, and performance in AI-driven work. Moreover, this study contributed to distinguishing AI implementation from more conventional digitalization or automation processes. Unlike earlier technologies that primarily affected operational routines, AI reshapes the cognitive and interpretive dimensions of work, influencing decision-making authority, task significance, and role identity. As such, AI implementation cannot be conceptualized as a purely technical process, but rather as a transformation that affects how employees understand and value their contribution within the organization. Generative AI systems often produce “hallucinations”, which are inaccurate outputs that appear plausible or true (Beutel et al., 2023). When employees lack adequate training to detect these limitations, they are at risk of making unreliable decisions that could compromise their job performance. In this regard, ECAII represented a strategic response that supports employees’ sensemaking and involvement during technological change, helping preserve and reconstruct meaningful experiences of work at a time when job content and professional identity may be shifting.

**Figure 1:**
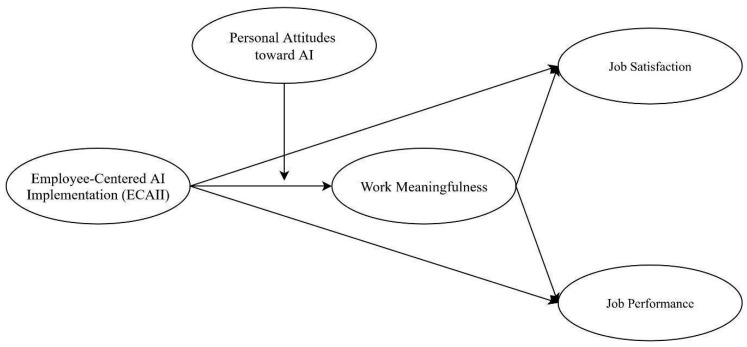
The research model.

Building on these considerations, the present study aimed to examine the processes and conditions through which ECAII contributed to positive employee outcomes. Specifically, we investigated whether and to what extent ECAII was related to job satisfaction and performance, both directly and indirectly through the mediating role of work meaningfulness. This latter variable was considered because it reflected the value employees see in their work in relation to their own ideals and standards (Hackman & Oldham, 1980; May et al., 2004) and represented a central psychological mechanism that organizations could foster by actively involving employees in their strategy, ultimately enhancing well-being and effectiveness. Moreover, we investigated whether the effects of ECAII may vary depending on employees’ attitudes toward AI, considering their interaction to bridge organizational and individual perspectives on new technology adoption. Employees’ personal attitudes toward AI were included as a moderator because they capture individual openness to technological change, which could amplify the effectiveness of organizational strategies such as ECAII. To test these relationships, as shown in the model presented in Figure 1, we used Structural Equation Modeling (SEM). Specifically, the mediating role of work meaningfulness and the moderating role of personal attitudes toward AI in the relationship between ECAII and the positive outcomes of job satisfaction and performance were tested.

## 2. Theoretical Background

### 2.1. Employee-Centered AI Implementation (ECAII) for Job Satisfaction and Performance

Moving from the assumptions drawn above, the study argued that employee-centered AI implementation (ECAII) was directly related to employees’ job satisfaction and performance. ECAII refers to organizational strategies that foster employees’ awareness, involvement, and support during the adoption of AI systems. This implementation strategy focused on three main practices: information, consultation, and training (González-Romá et al., 2025).

Information refers to the communication of clear and relevant knowledge that helps employees understand their work with AI, how it generates value, and how it fits into broader organizational processes. By providing information, organizations reduce ambiguity and hindrance stressors while also enhancing employees’ willingness and ability to respond effectively to work demands (LePine et al., 2005).

Consultation refers to inclusion of employees in decision-making processes related to AI change. It is important because employees often hold unique insights into workflows and processes that managers may overlook (Jones et al., 2010). When employees are consulted, they use their expertise and ideas to understand change, to make sense and accept decisions, finally increasing their sense of agency. When organizational information and technological changes are communicated, employees can better understand how their individual efforts align with the broader purpose. To support this alignment, organizations develop targeted training programs.

Training refers to organizational practices of learning and development aimed at equipping employees with the competencies required to face AI changes. Training initiatives are not limited to formal programs but also include opportunities to support informal learning, such as peer-to-peer exchanges and on-the-job development (Manuti et al., 2015). When employees are supported in developing their competencies with AI, they can gain greater confidence, reduce the negative feelings associated with uncertainty, and improve their performance.

Taken together, these practices compose ECAII, ensuring that AI implementation is not only technically effective but also aligned with employees’ experiences and needs in order to maximize the success of AI in the workplace. Importantly, ECAII was conceptualized not as the simple presence of isolated practices (e.g., providing training or communicating about AI), but as a coherent implementation philosophy that integrated communication, consultation, and competence development into a unified approach. This distinguished ECAII from more fragmented change practices and emphasizes that the effectiveness of AI implementation depended on the consistency and perceived intentionality of the strategy adopted by the organization. Consistent with HRM research emphasizing the importance of internally coherent bundles of practices (Boon et al., 2019), ECAII captured a unified approach to AI implementation that integrated practices that operate through complementary and mutually reinforcing psychological mechanisms during AI implementation. Information alone may enhance cognitive understanding, but without consultation it risks being perceived as top-down communication; consultation without adequate information may limit employees’ ability to contribute meaningfully; and training without a shared understanding of the rationale and goals of AI may be experienced as instrumental rather than empowering. It is the joint presence of these practices that enables employees to make sense of AI-related changes, feel legitimately involved in them, and develop the competencies needed to act effectively within AI-enabled work systems.

By actively considering the human side of technological change, such practices could enhance employees’ experiences of their work, leading to beneficial individual outcomes, such as higher job satisfaction and better performance (Nurlia et al., 2023; Petersson et al., 2022; Valtonen et al., 2025). Job satisfaction can be defined as an evaluative judgment about the job, reflecting the extent to which employees perceive their work as favorable, capturing a general attitude toward the job as a whole, or about specific facets such as tasks, pay, or opportunities (Judge et al., 2017). Job performance can be defined as the set of behaviors and outcomes reflecting both the quality and quantity of work, through which employees contribute to organizational goals (Campbell, 1990; Viswesvaran & Ones, 2000).

The link between ECAII and these positive outcomes was grounded in Lawler’s (1986) high involvement management framework and its further developments (Alqudah et al., 2022; Garmendia et al., 2021; Zhao et al., 2022), which posited that organizational practices that provide employees with information, resources, and opportunities to participate in decision-making enhance both motivation and effectiveness. Translated into the context of AI implementation, this perspective was particularly relevant because AI-based systems tend to increase informational complexity, alter decision-making processes, and redistribute control over work activities. Rather than being inherently detrimental, these changes could generate uncertainty and perceived loss of control when employees have limited access to information, limited opportunities for voice, or insufficient support to interpret and use AI outputs (Kellogg et al., 2020; Petersson et al., 2022). Under these conditions, high-involvement practices become critical mechanisms through which organizations can help employees interpret AI-related changes (Vishwakarma & Singh, 2023). Involving employees in the introduction of AI may therefore foster a better understanding of how intelligent systems operate and affect work roles, reduce ambiguity associated with algorithmic decision-making, and support employees’ sense of agency in AI-enabled work. By addressing these AI-specific challenges through information sharing, consultation, and skill development, high-involvement practices promote active engagement with AI systems. For example, organizations may provide transparent information about the purposes, functioning, and limitations of AI systems; allocate resources such as training, technical support, and time for experimentation; and offer opportunities to participate through consultation, feedback mechanisms, or involvement in the redesign of AI-supported tasks. Together, these processes foster a positive work experience, ultimately leading to higher levels of job satisfaction and performance.

In line with this reasoning, empirical research highlighted the beneficial role of high-involvement practices for employee positive outcomes. For example, Ahmad et al. (2014) found strong associations of high-involvement management with both job satisfaction and performance in the banking sector, stressing the importance of involving and empowering employees. Similarly, Wood et al. (2012) reported positive effects of high-involvement management practices (including information disclosure, skills training, functional flexibility, suggestion schemes, and team briefings) supporting the idea that organizational strategies centered on employees contribute to more positive experiences and results. More recently, Garmendia et al. (2021), examining the retail sector, emphasized the relevance of clear communication and quality information in times of uncertainty to reduce employee resistance. They also highlighted that, while immediate effects may be limited, long-term investments in high-involvement management practices are crucial, given their positive impact on employee job satisfaction over time.

In the context of AI implementation, only a limited number of studies investigated the impact of organizational strategies on job satisfaction and performance of employees working with AI. Among these recent studies, Nurlia et al. (2023) showed that AI implementation strategies can influence workforce productivity through training initiatives and the adaptation of organizational structures, cultures, policies, and processes to the changes introduced by AI. Similarly, Bergquist et al. (2024), drawing on qualitative interviews, emphasized the role of trust in AI among employees, noting that one of its key conditions relates to the organizational systems and practices supporting AI adoption. Petersson et al. (2022), also based on interviews in the healthcare sector, highlighted the need for a systematic approach to AI implementation, which requires an infrastructure for strategic change management combined with clear leadership capable of supporting the integration of AI into employees’ daily practice.

Considering the theoretical justifications and prior empirical evidence, we expected that ECAII was directly associated with positive employee outcomes because involving employees in AI implementation could foster positive work experiences with AI, enhancing their subjective evaluation of the job and their capacity to contribute effectively. Therefore, it was assumed that:

**Hypothesis 1.**  *ECAII was directly and positively related to job satisfaction (H1a) and performance (H1b).*

### 2.2. The Mediating Role of Work Meaningfulness

A second assumption of the present study was that work meaningfulness could mediate the relationship between ECAII and the positive outcomes of job satisfaction and performance. Work meaningfulness can be described as the perceived value of work objectives, as referred to personal ideals and standards (Hackman & Oldham, 1980; May et al., 2004). Meaningful work contributes to the achievement of an organization’s goals, enhancing employees’ sense of fulfillment at work. Organizational practices and communication of information play a pivotal role in aligning individual and organization’s aims, helping individuals understand how their contribution supports broader objectives. This alignment may become particularly critical during AI-driven change, as the introduction of intelligent systems can alter task content, decision authority, and perceptions of contribution, potentially undermining employees’ sense of purpose and value. Building on this perspective, the study argued that during times of AI-driven change, the value employees attribute to their work could be influenced by organizational practices that fostered involvement in the implementation process (i.e., ECAII). Such practices could strengthen employees’ connection to their work by helping them reinterpret their role and contribution within an AI-enabled context, thereby reinforcing work meaningfulness amid technological transformation.

The rationale underlying the mediating role of meaningful work was grounded in the Job Characteristics Model (Hackman & Oldham, 1980), which identified meaningfulness as a core psychological state that organizations could actively foster through the strategic design of work to enhance employees’ motivation and satisfaction. According to this theoretical perspective, work becomes meaningful when employees experience their tasks as significant and valuable, which in turn promotes positive outcomes. In the context of AI, this model remains highly relevant, as AI technologies directly reshape core job characteristics by redistributing tasks between humans and machines, redefining skill requirements, decision-making, and motivational processes. AI systems may reduce or expand skill variety, modify task identity by fragmenting or augmenting work activities, and influence perceived task significance by changing how human contributions relate to organizational outcomes. Moreover, the delegation of cognitive or decision-related tasks to AI can affect employees’ experienced responsibility and their understanding of how their actions influence results. Thus, the Job Characteristics Model offers a useful framework to explain why AI implementation may either undermine or enhance work meaningfulness, depending on how work is redesigned and how employees make sense of their evolving role within human–AI collaboration. During AI implementation processes, this perspective suggests that organizations can foster meaningfulness by adopting an ECAII strategy that highlights employees’ importance in the implementation process and recognizes their role as co-creators of change. Specifically, an ECAII strategy may enhance work meaningfulness in several ways. Clear information helps employees make sense of new tasks, consultation could enhance their sense of being part of organizational change, and training could boost their confidence in leveraging their competencies to add value within an AI-enabled workplace. Therefore, employees could attribute new value and significance to their work, resulting in higher job satisfaction and self-effectiveness in working with AI (Allan et al., 2019).

Prior research highlighted the effect of organizational strategies on the development of meaningful work, ultimately affecting positive work-related outcomes. For example, Landells and Albrecht (2019) showed the mediating role of work meaningfulness in the relationship between organizational politics and work engagement, underscoring its function as a key psychological mechanism explaining how organizational dynamics influence employee outcomes. Regarding the effects of organizational factors on work meaningfulness, Lysova et al.’ (2019) review highlighted that HR practices strategically designed to foster engagement can enhance employees’ psychological experience of meaningfulness (Albrecht et al., 2015; Pratt & Ashforth, 2003). Perceived opportunities for training and development have likewise been positively associated with meaningful work (Fletcher, 2016). A substantial body of research has documented the positive consequences of meaningfulness for job-related outcomes (Allan et al., 2019). For instance, Humphrey et al. (2007) found that task significance and meaningfulness contribute to higher individual effectiveness. Steger et al. (2012) showed that meaningful work is strongly associated with job satisfaction, and Lepisto and Pratt (2017) highlighted its role in motivating performance and proactive work behaviors.

In the context of AI, recent studies examined the implications of AI introduction for employees’ experiences of meaningful work. Conceptual contributions suggested that AI has the potential to both enrich and undermine meaningfulness at work, depending on how it affects task significance, autonomy, and opportunities for skill use (Bankins & Formosa, 2023; Knell & Rüther, 2024; Smids et al., 2020). These perspectives emphasized that meaningful work in the age of AI is not automatically ensured, but rather depends on how technological changes are managed and integrated within organizational practices. Consistent with this line of reasoning, Callari and Puppione (2025) provided empirical insights showing that employees’ perception of meaningful work after the introduction of AI in their work evolved through the interplay between organizational arrangements and employees’ subjective perceptions of AI’s role at work. Taken together, this evidence suggested that organizational strategies of AI implementation, such as ECAII, may play a central role in influencing how employees make sense of their work with AI, which in turn influences their job satisfaction and effectiveness at work.

Considering the theoretical justifications and empirical evidence presented above, it was expected that employee exposure to ECAII would have a positive indirect effect on job satisfaction and performance via work meaningfulness. ECAII practices that provide employees with transparent information, active involvement, and opportunities for training were likely to foster the perception of work as more meaningful, which, in turn, would promote higher satisfaction and effectiveness at work. Therefore, the study assumed that:

**Hypothesis 2.**  *ECAII was indirectly and positively related to job satisfaction (H2a) and performance (H2b) through the mediation of work meaningfulness.*

### 2.3. The Interaction Between Employee-Centered AI Implementation (ECAII) and Personal Attitudes Toward AI

The effectiveness of an organizational strategy depends not only on the practices it encompasses (e.g., information, consultation, and training) but also on the characteristics and attitudes of the employees who are involved in the strategy and whose work is influenced by that change. Personal attitudes toward AI can be described as individuals’ general evaluations, predispositions, and openness toward the use of AI technologies at work (Lichtenthaler, 2019). They represent the individual side of the AI implementation process and are therefore crucial to consider in interaction with organizational practices. Personal attitudes shape how employees perceive and respond to AI implementation. Thus, we posited that the relationship between employee-centered AI implementation (ECAII) and work meaningfulness was moderated by employees’ personal attitudes toward AI. Specifically, the positive effect of ECAII on work meaningfulness may vary depending on employees’ predispositions toward AI. We expected that this relationship would be stronger among employees with favorable attitudes, as they could be more likely to interpret AI-related initiatives as congruent with their personal orientations and beneficial for their work.

The rationale for the moderating role of personal attitudes was grounded in the Congruence Theory (Nadler & Tushman, 1980), which emphasized that organizational effectiveness depends on the degree of alignment between the key components of an organization, including tasks, people, structure, and culture. When the components are congruent, organizations are more likely to achieve positive outcomes. Applied to the AI context, this framework suggested that the success of AI implementation depended on the alignment between organizational practices (e.g., information, consultation, and training within ECAII) and the employees’ characteristics, such as their attitudes toward AI. When employees hold positive attitudes, the implementation initiative could be perceived as acceptable and supportive, thereby fostering work meaningfulness and, in turn, job satisfaction and performance. Conversely, when employees have negative attitudes, a misfit may emerge, so that even well-designed AI implementation strategies could be met with resistance, reducing their potential to enhance meaningfulness and its outcomes.

In this perspective, prior studies highlighted that the impact of organizational factors, such as strategies and practices, on work-related outcomes may depend on the individual characteristics and attitudes of employees. For example, Lysova et al. (2019) highlighted the importance of the interaction between organizational and individual factors in fostering meaningful work by reviewing studies on the antecedents of employees’ experiences of meaningfulness. Wang and Xu (2017) showed that ethical leadership was more effective in fostering employees’ meaningfulness work when employees had higher core self-evaluations. In the context of AI implementation, Zhang et al. (2025) found that openness to experience moderates the impact of AI usage on self-efficacy, such as those individuals with higher openness benefited more from AI introduction in their work, which in turn positively influenced their innovation behavior in employees. Shweta and Panicker (2025) found a significant interaction effect between leadership and employees’ attitudes toward AI on work meaningfulness, underscoring the importance of organizational strategies that acknowledge and lead individuals’ predispositions to foster meaningful work.

Building on this reasoning and extending the indirect effect proposed in Hypothesis 2, it was proposed that personal attitudes toward AI could moderate the indirect relationship between ECAII and employee outcomes. Specifically, employees’ attitudes were expected to shape the extent to which ECAII practices foster work meaningfulness and, in turn, enhance job satisfaction and performance. Therefore, it was argued that:

**Hypothesis 3.**  *The indirect positive relationships between ECAII and job satisfaction (H3a) and performance (H3b) were moderated by personal attitudes toward AI, so that these indirect effects were stronger when personal attitudes were high and weaker when personal attitudes were low.*

## 3. Method

### 3.1. Procedure and Participants

Data were collected through an online survey administered to Italian employees between November 2024 and February 2025, using a non-probability, convenience sampling approach. The study adopted a cross-sectional research design. To be eligible for participation, respondents had to be currently employed and have experienced changes in their work activities or tasks due to the introduction of AI technologies in their organization.

The research was conducted within the framework of a broader project on workplace automation funded by the European Union. The study complied with ethical standards and relevant data protection regulations and received approval from the Ethics Committee of the authors’ university. The questionnaire included socio-demographic questions as well as validated scales measuring the key study variables, namely Employee-Centered AI Implementation Practices (ECAII), personal attitudes toward AI, work meaningfulness, job satisfaction, and job performance. Participants were provided with a short description of the study’s objectives and were required to provide informed consent electronically. Participation was entirely voluntary and anonymous, and no monetary or material incentives were offered.

An a-priori power analysis for SEM was conducted using the RMSEA-based approach with α = 0.05, power = 0.90, and 162 degrees of freedom, corresponding to the hypothesized model with 20 observed variables and five latent factors. The analysis indicated that a minimum sample size of 151 participants was required to ensure adequate statistical power. The final sample that was collected consisted of 168 white-collar employees actively using AI in their work. Participants’ mean age was 35.38 years (*SD* = 11.88), and 43% were women. Regarding educational attainment, 75% held a university degree, while 25% had completed secondary education. Regarding company size, 27% of participants worked in small enterprises (fewer than 50 employees), 17% in medium-sized companies (51–250 employees), and 56% in large organizations (more than 250 employees) operating in the private (79%) and public (21%) sectors. All participants were employed in the tertiary (services) sector, ensuring a degree of sectoral homogeneity in the sample. Additional details on the demographic variables are reported in Supplementary Table S1.

### 3.2. Measures

Employee-Centered Artificial Intelligence Implementation (ECAII). ECAII practices were assessed using seven items adapted from González-Romá et al. (2025), originally developed to capture employee-centered automation implementation strategies in organizational change contexts. The scale refers to the extent to which organizations adopt implementation practices that actively inform, consult, and involve employees during the introduction of automation systems. Items were adapted to specifically refer to AI implementation. Responses were given on a 6-point Likert scale ranging from 1 (strongly disagree) to 6 (strongly agree). An example item is “Employees were informed about the main reasons behind the AI implementation”. Internal consistency was good (α = 0.94). Items were adapted through a systematic wording substitution procedure to refer specifically to AI implementation (e.g., replacing “automation” with “AI systems” and specifying the context of introduction). The adaptation followed a meaning-preservation approach to ensure conceptual equivalence with the original scale while increasing contextual relevance.

Personal attitudes toward AI. Employees’ attitudes toward AI were measured using four items adapted from Nomura et al. (2006), which assess individuals’ general evaluations and openness toward technologies in the workplace. The scale captures the degree to which employees perceive AI as useful and beneficial for their work. Responses were provided on a scale ranging from 1 (strongly disagree) to 6 (strongly agree). An example item is: “AI facilitates employees’ work activities”. Internal consistency was good (α = 0.88).

Work meaningfulness. This variable was measured with three items from May et al. (2004), assessing the perceived significance and purposefulness of one’s work. Responses were given on a scale ranging from 1 (strongly disagree) to 6 (strongly agree). An example item is: “The work I do on this job is worthwhile”. Internal consistency was good (α = 0.95).

Job satisfaction was measured using three items from González-Romá and Hernández (2016), concerning an evaluative judgment about the job which reflects the extent to which employees perceive their work as favorable. Responses were given on a scale ranging from 1 (very dissatisfied) to 6 (very satisfied). An example item is: “Indicate how dissatisfied/satisfied you are with the work you do”. Internal consistency was acceptable (α = 0.76).

Job performance. This variable was measured using three items adopted by Kampf et al. (2021) which were originally derived from previous measures (Bond & Bunce, 2001; Welbourne et al., 1998). The scale assessed employees’ self-rated performance over the previous week, capturing their perceived effectiveness and quality of work outcomes. Responses were given on a scale ranging from 1 (very low) to 5 (very high). An example item is: “How would you evaluate the overall quality of your work last week?”. Internal consistency was good (α = 0.79).

### 3.3. Data Analysis

All analyses were conducted using Mplus 8 (Muthén & Muthén, 2017). The hypothesized model involved direct, indirect, and moderated relationships between latent constructs. Prior to testing the structural model, we evaluated the multivariate normality for the observed indicators using Henze and Zirkler’s (1990) test, which indicated a violation of this assumption (1.08, *p* < 0.001). The lack of multivariate normality could contribute to discrepancies across different fit indices, such as a significant chi-square test alongside acceptable comparative and parsimonious fit indices, which should therefore be interpreted with caution. As a result, we applied the Robust Maximum Likelihood estimator (MLR) in the subsequent structural equation modeling (SEM) analyses.

We conducted a series of confirmatory factor analyses (CFA) to assess the adequacy of the measurement model. The hypothesized six-factor model, corresponding to the six latent constructs included in the structural model, showed an acceptable fit to the data (χ^2^(158) = 282.98, *p* < 0.05; CFI = 0.94; TLI = 0.93; RMSEA = 0.07; SRMR = 0.06) and the detailed standardized factor loadings are reported in the Supplementary Table S2. As a robustness check, we estimated a one-factor model (Harman’s single-factor test) in which all items were constrained to load onto a single latent factor. The fit indices for this alternative model were poor (χ^2^(170) = 1300.59, *p* < 0.001; CFI = 0.43; TLI = 0.36; RMSEA = 0.20; SRMR = 0.18), confirming that a single-factor structure could not account for the observed data.

To test the direct and indirect effects posited in Hypotheses 1 and 2, we estimated a structural model including both direct paths from ECAII to satisfaction and performance, as well as indirect paths via work meaningfulness. This model was compared with a more constrained version in which the direct paths were fixed to zero, using a chi-square difference test. To evaluate the significance of the indirect effects, we computed bootstrap 95% confidence intervals with 5000 resamples.

Hypothesis 3 involved a latent moderation mechanism, in which personal attitudes toward AI were expected to moderate the relationship between ECAII and work meaningfulness, and subsequently the indirect effects on the outcomes. Because SEM models including latent interactions involve non-linear terms, conventional fit indices are not reliable and are not provided by Mplus (Kelava et al., 2011). Thus, to assess model fit, the full model including the latent interaction term was compared with a model excluding the interaction, using a log-likelihood chi-square difference test with corresponding scaling correction factors (Cheung et al., 2021). To examine the role of the interaction on the indirect effects, we used bootstrap estimation and calculated estimates at ±1 standard deviation of the moderator. Finally, we employed Johnson–Neyman plots to visualize the range of moderator values for which the indirect effects were statistically significant.

## 4. Results

Descriptive statistics, internal consistency coefficients, and bivariate correlations for all variables included in the study were summarized in Table 1. As shown, all correlations were statistically significant and in the expected positive direction, providing preliminary support for the hypothesized direct relationships among the study variables. However, while the correlation coefficients were statistically significant, they explain a relatively low amount of variance. This pattern could reflect the relatively small sample size and the complexity of the relationships among the study constructs. Accordingly, the correlations reported as well as the subsequent model-based results should be interpreted with caution, as statistical significance alone could overstate the substantive strength of the observed associations.

**Figure 2:**
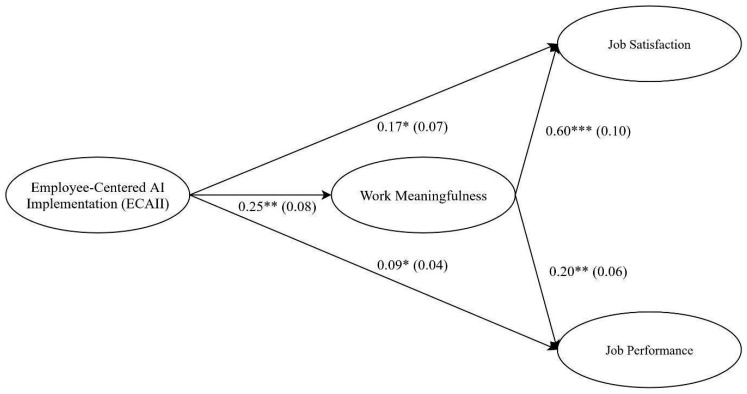
Parameter estimates for the latent mediation model. Note. * *p* < 0.05; ** *p* < 0.01; *** *p* < 0.001. Coefficients were unstandardized. Standard errors were shown within parentheses. Indicators were not shown for the sake of clarity.

To test the hypothesized direct and indirect effects (H1 and H2), we first examined a model including both the direct paths from Employee-Centered AI Implementation (ECAII) to job satisfaction and performance, and the indirect paths through work meaningfulness. The model demonstrated an acceptable fit to the data (χ^2^(96) = 214.68, *p* < 0.001; CFI = 0.93; TLI = 0.91; RMSEA = 0.08; SRMR = 0.06), with statistically significant path coefficients, as shown in Figure 2, which reported the estimated unstandardized direct effects. Specifically, ECAII had significant direct effects on both job satisfaction (B = 0.17, SE = 0.07, *p* < 0.05) and job performance (B = 0.09, SE = 0.04, *p* < 0.05), as hypothesized (H1). Moreover, the bootstrapping procedure showed that the indirect effects of ECAII via work meaningfulness were statistically significant for both job satisfaction (B = 0.15, SE = 0.06, 95% CI [0.04, 0.25]) and job performance (B = 0.05, SE = 0.02, 95% CI [0.02, 0.09]), providing support for Hypotheses 2a and 2b. The calculation of the completely standardized indirect effects indicated effect sizes of 0.18 and 0.12 for the indirect effects on job satisfaction and performance, respectively.

To confirm that the inclusion of direct effects improved model fit, we compared this model with a more constrained model in which the direct paths from ECAII to job satisfaction and performance were fixed to zero. The comparison revealed a significantly better fit for the model including direct paths, Δχ^2^(2) = 10.32, *p* < 0.01, indicating that, beyond the indirect effects via work meaningfulness, ECAII had unique associations with the outcome variables.

**Figure 3:**
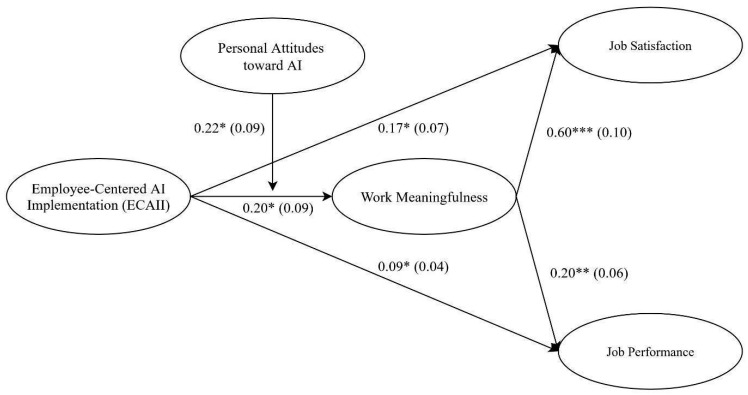
Parameter estimates for the latent moderated mediation model. Note. * *p* < 0.05; ** *p* < 0.01; *** *p* < 0.001. Coefficients were unstandardized. Standard errors were shown within parentheses. Indicators were not shown for the sake of clarity.

To test Hypotheses 3a and 3b regarding the moderating role of personal attitudes toward AI, moderated mediation analysis using latent interaction modeling was conducted. In a preliminary model, personal attitudes toward AI and its main effect on work meaningfulness were included, but the latent interaction term was excluded. This model was used to retrieve fit indices, since models with latent interactions yield unreliable fit statistics (Kelava et al., 2011), showing a satisfactory fit to the data (χ^2^(160) = 291.92, *p* < 0.001; CFI = 0.93; TLI = 0.92; RMSEA = 0.07; SRMR = 0.07). Subsequently, a model including the latent interaction between ECAII and personal attitudes on work meaningfulness was estimated. A chi-square difference test confirmed that the model with the interaction term fit significantly better than the one without it, Δχ^2^(1) = 6.61, *p* < 0.01, confirming the importance of the interaction effect. Figure 3 displayed the unstandardized parameter estimates for the model including the latent interaction. The interaction effect was significant (B = 0.22, SE = 0.09, *p* < 0.05), indicating that the relationship between ECAII and work meaningfulness varied depending on employees’ attitudes toward AI.

**Figure 4:**
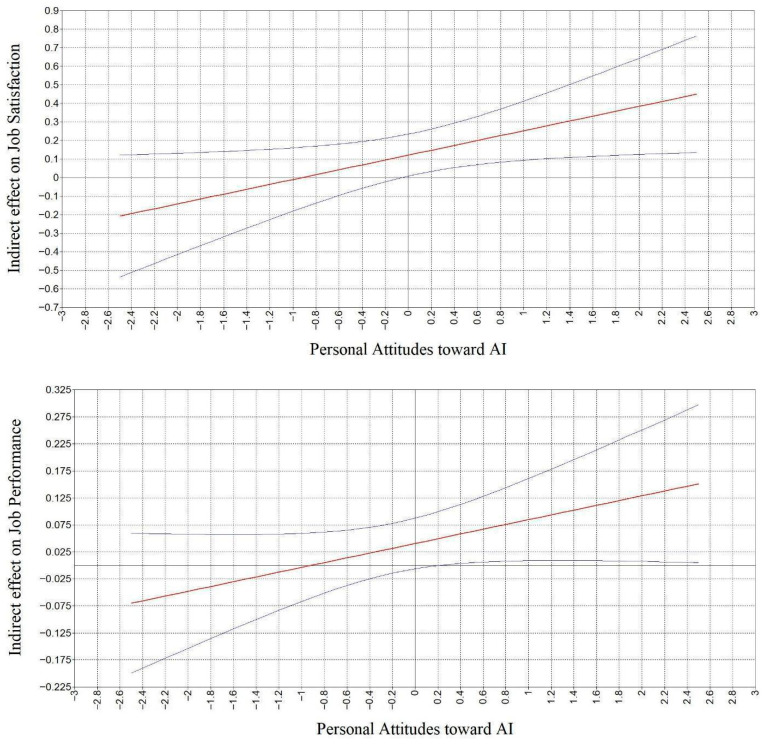
Plot of the conditional indirect effects of Employee-Centered Artificial Intelligence Implementation (ECAII) on Job Satisfaction (**top panel**) and Job Performance (**bottom panel**) across values of Personal Attitudes toward AI. Note. The distribution of the moderator was mean-centered. The red lines represented the point estimates of the indirect effects across the range of Personal Attitudes toward AI, the blue lines indicated the corresponding 95% confidence intervals.

Specifically, to examine whether the significant interaction also affected the indirect effects of ECAII on the two outcomes, we computed the conditional indirect effects (Preacher et al., 2007) through work meaningfulness at different levels of the moderator (±1 SD). The results were reported in Table 2 showing that at high and mean levels of personal attitudes toward AI, both indirect effects were positive and statistically significant, with stronger effects observed at high levels of the moderator. At low levels of the moderator, neither indirect effect reached statistical significance. To better interpret these variations in indirect effects depending on the moderator, we adopted Johnson–Neyman plots (see Figure 4), which showed that the strength of the indirect effects increased with higher levels of personal attitudes toward AI. Taken together, these findings supported Hypotheses 3a and 3b.

## 5. Discussion

The aim of this study was to examine how organizational practices of Employee-Centered AI Implementation (ECAII) were related to employee outcomes, shedding light on the underlying psychological mechanisms and individual conditions involved. To address this aim, a survey was conducted with a sample of employees who had experienced AI-related changes in their tasks and work activities, and a series of latent models were tested to examine the mediating role of work meaningfulness and the moderating role of personal attitudes toward AI in the relationship between ECAII, job satisfaction, and performance. Results confirmed all the hypotheses of the study.

Hypothesis 1 was supported, as ECAII showed significant direct associations with both job satisfaction (H1a) and performance (H1b). The positive relationship with job satisfaction highlighted the importance of organizational strategies that actively involve employees in the strategic implementation of AI. By providing information and guidance, organizational strategies help employees to interpret technological changes as beneficial, understand how their contribution aligns with organizational objectives, and experience greater satisfaction in AI-enabled contexts. Similarly, the significant positive association with job performance suggested that employee-centered implementation strategies may not only shape employees’ well-being but may also enhance their effectiveness and productivity. These findings extended previous research by showing that the benefits of involving employees in technological implementation were not limited to well-being outcomes but also translated into improved self-rate performance indicators. This result aligned with evidence indicating that well-designed HR and change management practices could affect organizational and individual performance (Ahmad et al., 2014; Lawler, 1986; Nurlia et al., 2023).

Hypothesis 2 was supported, as ECAII practices were indirectly related to both job satisfaction and job performance through employees’ perceptions of work meaningfulness. Although the indirect path to job performance showed a smaller magnitude, these findings underscored that work meaningfulness represents a crucial psychological mechanism through which HR practices translate into positive employee outcomes, clarifying not only that these practices matter but also why they are effective. By fostering meaningful work, organizations could help employees to enhance individual fulfillment at work and organizational aims. This mechanism becomes particularly relevant in the context of AI, where the meaning of work may shift due to changes in job characteristics brought about by cognitive automation (Hackman & Oldham, 1980). Previous studies suggested that the introduction of AI may threaten employees’ sense of meaningfulness, for instance when automation reduces their discretion, autonomy, or perceived contribution (Knell & Rüther, 2024; Smids et al., 2020). At the same time, AI can also provide opportunities to create new sources of meaning, for example, by freeing employees from repetitive tasks and allowing them to focus on more complex, creative, and valuable activities (Bankins & Formosa, 2023; Smids et al., 2020). From this perspective, employee-centered strategies such as ECAII may help co-construct new meanings with employees, using practices that ensure AI implementation enhances rather than undermines the meaningfulness of work. By linking employees’ tasks to organizational objectives in a way that is understandable, these strategies strengthen positive outcomes for employees, including satisfaction and performance.

Hypothesis 3 was supported, as employees’ personal attitudes toward AI moderated the relationship between ECAII practices and work meaningfulness, with consequences on the indirect effects on job satisfaction and performance. This means that employee attitudes could amplify the effectiveness of organizational strategies, shaping how employees make sense of their work in the context of AI-driven change. These results pointed to the importance of considering the interaction between organizational and individual perspectives, because when organizational strategies and personal orientations toward AI are aligned and congruent (Nadler & Tushman, 1980), employees are more likely to construct meaningful experiences at work, which in turn enhances their satisfaction and effectiveness.

### 5.1. Theoretical Implications

This study aligned with existing research on High Involvement Management and meaningful work, but extended these frameworks to the context of AI, where the introduction of technology involves changes not only in work tasks but also in how employees interpret and relate to their roles. In the case of cognitive automation, these changes are particularly critical, as AI systems may alter or displace traditional sources of work meaningfulness, such as task ownership, professional judgment, and the perceived social value of one’s contribution. However, our findings pointed out that the process of redefining work meaningfulness is shaped by how organizations manage AI implementation to actively involve people rather than by AI technology implementation itself. The key theoretical contribution lied in showing that employee involvement practices (e.g., information, consultation, and training) were means of sustaining and co-constructing the meaningfulness of work in contexts characterized by cognitive automation. In doing so, the study shifted the focus from technological adoption to the subjective, interpretive processes through which employees make sense of organizational change, highlighting that the success of AI implementation depends on how individuals reformulate the purpose and value of their work.

Findings offered several contributions to theory. First, they contributed to HRM and organizational change research (Alqudah et al., 2022; Lawler, 1986; Zhao et al., 2022) by highlighting the role of employee-centered implementation practices as strategic levers in the age of AI. High involvement approaches emphasized that organizations could achieve superior outcomes by systematically sharing information, developing employee skills, and empowering participation in decision-making. By transferring these principles to the context of AI, the study findings showed that transparent communication, broad consultation, and targeted training (i.e., ECAII practices) could contribute to creating a participatory environment that strengthens employees’ understanding, motivation, and ability to contribute to the success of technological change. In this way, ECAII acted as a bridge between organizational strategy and employee experiences, fostering conditions that support more favorable employee outcomes at work. Thus, the novelty of the role of ECAII lied in creating the structural and relational conditions that enabled employees to reinterpret the purpose and value of their work in light of AI-driven change, particularly as tasks, responsibilities, and decision-making processes were reshaped by cognitive automation. In this sense, ECAII supported employees’ sensemaking processes, enabling the reconstruction of work meaning when established role boundaries and expertise were challenged by AI.

Second, results coming from the study advanced research on meaningful work by identifying it as a key psychological mechanism that links HR practices to employee outcomes during technological transformations. Classic theories such as the Job Characteristics Model (Hackman & Oldham, 1980) posited that core job characteristics influence employees’ meaningfulness, which in turn influences their internal motivation and effectiveness at work. Our results extended these principles to AI contexts, showing that organizational strategies involving employees in AI implementation could help preserve or enhance meaningfulness even as job characteristics evolve due to automation. Specifically, ECAII practices provide employees with information to make sense of new tasks, opportunities to voice their perspectives, and training to develop competencies aligned with emerging technologies. This fosters a sense of purpose and value in their work, which then translates into more positive attitudes and behaviors. From this perspective, our findings contributed to empirical evidence on AI implementation, highlighting that effective collaboration between humans and AI meets employees’ fundamental and growth needs related to the overall quality of their work life (Wu et al., 2025). Moreover, building on previous studies showing that promoting organizational cultures that value innovation and transparency can foster confidence in AI systems among employees (Olaitan et al., 2021), our study advanced this field by identifying work meaningfulness as a key mechanism through which the interaction between ECAII practices and employees’ attitudes towards AI resulted in positive outcomes such as job satisfaction and job performance.

Third, this research underscored the relevance of person–organization congruence in shaping employees’ experiences during AI-driven transformations. Drawing on Congruence Theory (Nadler & Tushman, 1980), we showed that the alignment between organizational practices and employees’ personal attitudes toward AI was critical for fostering meaningfulness and promoting positive outcomes. Considering both the organizational and individual perspectives on AI allowed us to understand how similar implementation strategies could lead to different employee reactions. When organizational practices that promote involvement were matched by positive individual orientations toward AI, employees were more likely to attribute significance to their changing roles and embrace AI as a resource rather than a threat. This alignment strengthened the psychological impact of ECAII and maximized the potential benefits of AI strategies.

Overall, the theoretical contribution of this study did not consist in reaffirming the general importance of work meaningfulness for employee outcomes, but in explicating how meaningfulness is sustained by the organization in AI-driven work transformations. Specifically, the study showed that employee-centered AI implementation processes (referred to high-involvement practices of information, consultation, and training), in interaction with employees’ attitudes toward AI, could create the conditions through which individuals reinterpret the purpose and value of their work when established tasks, roles, and expertise are reshaped by cognitive automation.

### 5.2. Practical Implications

This study also yielded relevant practical implications for organizations implementing AI. The practical implications of this study could be particularly relevant for organizations facing AI-driven work transformations, where the introduction of cognitive automation altered work tasks, role boundaries, and decision authority.

First, consistent with our results showing a direct positive effect of ECAII on job satisfaction and performance, the evidence-backed findings indicated that transparent communication, active involvement, and targeted training fostered alignment between organizational goals and employees’ perceptions, increasing the likelihood that AI implementation would generate positive outcomes for both employees and organizations. These practices help reduce uncertainty and empower employees to see their role as central to the transformation process. Rather than approaching AI adoption as a purely technological or top–down change, organizations should treat it as a participatory process aimed at helping employees actively interpret how AI reshapes their contribution and value within the organization. This involves informing and consulting employees and supporting them through targeted training to enable adaptation to evolving work contexts. This implication was directly supported by the observed effects of ECAII on employee outcomes.

Second, building on the significant mediating role of work meaningfulness identified in our results, the evidence-backed findings of the study showed that supporting the development of meaningful work experiences emerged as a central strategy to sustain employee satisfaction and performance during AI changes. Organizations should view work meaningfulness not as a generic well-being dimension, but as a critical implementation lever that could enable employees to remain engaged and effective when AI could disrupt established work meanings. This involves deliberately designing interventions that help employees make sense of technological changes and connect them to their values and contributions. For example, organizations can promote participatory workshops where employees reflect on how AI alters their tasks and how their expertise remains crucial. Moreover, they can create spaces for employees to share experiences and reinterpret their roles in light of technological transformation. Leadership may also play a critical role by articulating a clear vision of how AI supports the organization’s mission and by linking employees’ work to organizational goals, thereby reinforcing the sense of purpose and significance. In this respect, leadership behaviors that focus on guiding sensemaking processes, ensuring ethical oversight, and aligning AI initiatives with organizational strategy appear particularly relevant for supporting employee-centered AI implementation, as AI reshapes decision-making, authority, and responsibility within organizations (Madanchian et al., 2024). Such leadership may be exercised not only by formal leaders but also through distributed roles across managerial and professional levels. These recommendations were aligned with our empirical evidence showing that ECAII enhanced satisfaction and performance through work meaningfulness.

Third, our findings showing that personal attitudes toward AI strengthened the indirect effects of ECAII suggested that implementation strategies were more effective when they resonated with employees’ orientations toward AI. At this stage, the following implications should be interpreted as prospective managerial considerations rather than direct empirical conclusions. Employees may differ in their openness, curiosity, or skepticism toward AI, and acknowledging these differences allows organizations to personalize implementation efforts. Several practical actions can foster this alignment. At earlier stages, organizations may adapt their recruitment and selection processes to attract individuals whose orientations toward technology fit their strategic goals, for example, by assessing digital openness or learning orientation. For existing employees, training programs can be designed not only to build technical competencies but also to address attitudinal aspects, by demystifying AI, showcasing its benefits, and encouraging critical and constructive dialogue. Targeted communication campaigns can be differentiated according to employee groups, addressing specific concerns and highlighting relevant opportunities. Moreover, feedback and consultation mechanisms can be used to systematically collect employees’ perspectives. Such initiatives help create a dynamic alignment between organizational goals and individual orientations, strengthening the efficacy of ECAII strategies.

Finally, in line with the central role of work meaningfulness emerging from our results, another prospective managerial consideration referred to the importance of complementing technical training with structured opportunities for shared sensemaking. Organizations can support employees by creating recurring spaces for reflection and dialogue that encourage individuals to reinterpret their role within the new AI-enhanced work environment. For example, guided role-redefinition workshops, peer discussion groups, or supported job crafting interventions can help employees articulate how their expertise continues to contribute to organizational goals. These initiatives do not replace skill development, but enhance it, transforming AI implementation from a top–down directive into a participatory and meaningful process. In addition, the study once again underscores the close interdependence between the individual and organizational dimensions. Specifically, even when discussing the implementation of AI within work and organizational practices, it is necessary to address the individual psychological dimension—in terms of the meaning of work, attitudes toward AI, and involvement—in order to achieve positive organizational outcomes in terms of performance and thus ensure the effectiveness of AI implementation within the organization.

Overall, these practical implications underscored that effective AI implementation could not be achieved through technical solutions alone. Organizations need to adopt coherent and structured implementation strategies that are aligned with their vision and mission, supported by HR practices oriented toward workforce involvement and listening, transparent communication, and training that respond to evolving needs. A particularly relevant insight emerging from this study concerned the central role of work-related sensemaking processes. Our results indicated that organizational involvement practices could support employees in reinterpreting and reconstructing the meaning of their work during AI-driven change so that they could accept and engage with the transformation. This process of work re-signification appears to be a key mechanism through which AI implementation could translate into higher well-being and performance.

### 5.3. Study Limitations and Future Research

This study has some limitations that should be considered when interpreting the results. First, the sampling strategy was non-probabilistic, and the sample is not representative of the entire Italian workforce. In detail, participants in the study were 168 white-collar employees working in the tertiary (services) sector and actively using AI in their work, mostly holding a university degree and primarily employed in large organizations, with a majority working in the private sector. These features could limit the generalizability of the findings. Future studies could replicate this research with more heterogeneous and representative samples, including employees from different occupational groups and sectors, to assess the robustness of our model across various work contexts.

Second, all variables were assessed through self-report questionnaires, which may raise concerns regarding common method variance and potential inflation of observed associations. Nonetheless, confirmatory factor analyses supported the distinctiveness of the constructs, and a single common-method factor did not account for the relationships observed among latent variables. Future studies could address this limitation by including additional data sources. In this regard, job performance results should be interpreted with caution. Due to calibration bias, employees may inflate their self-assessments of performance (Harris & Schaubroeck, 1988). Moreover, self-reported performance is not sufficient to draw conclusions about organizational effectiveness (Singh et al., 2016). Future research should address this limitation by using other-rated performance data. For example, supervisors could provide ratings of employees’ job performance, or objective performance indicators could be used to complement self-reported data.

Third, replacing ‘automation’ with ‘AI’ in our study’s measurement scales (e.g., the ECAII practices scale) may not capture specific nuances or variations in AI, such as generative AI or handling unstructured data. This could have limited the scale’s ability to represent AI’s distinctive features. To address this issue, future studies should develop specific AI measurement scales.

Fourth, the study relied on a cross-sectional design, which does not allow us to draw causal conclusions about the directionality of the observed relationships. Although the hypothesized paths were theoretically grounded, future longitudinal research is needed to examine how employee-centered AI implementation strategies, work meaningfulness, and employee outcomes unfold over time.

Fifth, our sample was focused on Italian white-collar employees, in light of the Italian government’s commitment to fostering AI development in work organizations (Agenzia per l’Italia Digitale, 2024). However, the lack of a comparison with different cultural contexts limits the generalizability of our findings. Future studies should adopt a cross-cultural perspective to deepen the understanding of how workers perceive AI implementation and its interaction with HR practices. This would enable us to examine whether these relationships vary across different cultural contexts.

A further direction for future research concerns the qualitative exploration of the interpretive processes through which employees reconstruct meaning in their work following AI implementation. Although the present study identified work meaningfulness as a mediating mechanism, the narrative, experiential, and relational dynamics through which meaning is negotiated remain underexplored. Longitudinal or qualitative designs could examine how ECAII functions as an ongoing relational infrastructure that shapes the evolution of work meaning over time in AI-enabled environments.

Finally, while the present study focused on personal attitudes toward AI as a key individual moderator, other individual differences related to employees’ coping strategies toward AI could be considered in further research. Future studies could explore the role of additional personal factors, such as digital skills, learning orientation, or occupational identity, in shaping the effectiveness of AI implementation strategies. Additionally, our findings should be extended by investigating the role of leadership styles (e.g., ethical, transactional) as organizational boundary conditions (Madanchian et al., 2024). Future studies could examine how different leadership approaches (e.g., strategic, ethical, or sense-giving leadership) and different sources of leadership (formal leaders vs. distributed leadership) interact with employee-centered AI implementation practices to shape work meaningfulness and performance outcomes.

## 6. Conclusions

This study advanced the understanding of how HR practices could foster positive employee experiences and outcomes in the context of AI-driven change. By integrating Employee-Centered AI Implementation (ECAII) strategies with psychological and individual perspectives, work meaningfulness was identified as a key mediating mechanism and personal attitudes toward AI was identified as a relevant boundary condition. Results highlighted that involving employees through transparent communication, consultation, and training could represent a strategic lever to sustain positive work experiences and effectiveness during technological transformation. Moreover, findings showed that the success of AI implementation depended not only on organizational strategies but also on their alignment with employees’ perspectives. Overall, this study contributed to bridging organizational change and meaningful studies, offering theoretical and practical insights into how organizations could foster beneficial outcomes for employees and organizations in the age of AI.

## Figures and Tables

**Table 1:** Descriptive statistics, Cronbach’s alpha coefficients, and Pearson’s correlations associated with the study variables.

Variables	M	SD	Skew.	Kurt.	1	2	3	4	5
1. Employee-Centered Artificial Intelligence Implementation (ECAII)	3.81	1.37	−0.24	−0.73	[0.94]				
2. Personal attitudes toward AI	4.63	1.02	−0.59	−0.36	0.33 ***	[0.88]			
3. Work meaningfulness	4.91	1.09	−0.74	−0.30	0.27 ***	0.18 *	[0.95]		
4. Job satisfaction	4.13	1.12	−0.49	−0.32	0.33 ***	0.32 ***	0.50 ***	[0.76]	
5. Job performance	3.80	0.75	−0.69	0.88	0.28 ***	0.31 ***	0.46 ***	0.54 ***	[0.79]

Note. N = 168. * *p* < 0.05; *** *p* < 0.001. M = Mean. SD = Standard Deviation. Skew. = Skewness. Kurt. = Kurtosis. Cronbach’s alpha coefficients were reported on the diagonal within brackets.

**Table 2:** Bootstrap confidence intervals for the conditional indirect effect of ECAII on job satisfaction and job performance via work meaningfulness at different levels of employees’ attitudes toward AI.

Moderator Values	Conditional Indirect Effect on Job Satisfaction			Conditional Indirect Effect on Job Performance		
	95% Lower Limit	Estimate	95% Upper Limit	95% Lower Limit	Estimate	95% Upper Limit
W mean − 1 SD	−0.317	−0.097	0.122	−0.108	−0.033	0.043
W mean value	0.014	0.121	0.227	0.001	0.041	0.081
W mean + 1 SD	0.128	0.339	0.551	0.025	0.114	0.203

Note. W = moderator variable (personal attitudes toward AI); SD = standard deviation. The conditional indirect effects were unstandardized.

## Data Availability

The data that support the findings of this study are available from the corresponding author upon reasonable request.

## Supplementary Materials

The following supporting information can be downloaded at: https://www.mdpi.com/article/10.3390/bs16020238/s1, Table S1. Demographic and Organizational Characteristics of the Sample. Table S2. Standardized Factor Loadings from Confirmatory Factor Analysis (CFA).
