# Supplementary material for: Making Artificial Intelligence Work at Work: The Role of Human Resource Practices and Personal Attitudes in Fostering Meaningful Work with Artificial Intelligence
Source: Behav Sci (Basel). 2026 Feb 8;16(2):238. doi: 10.3390/bs16020238 (PMC12938308; doi:10.3390/bs16020238)
Supplement: Supplementary file 1 [file behavsci-16-00238-s001.zip › behavsci-4078654-supplementary.pdf]

Supplementary Material

Table S1. Demographic and Organizational Characteristics of the Sample.

Variable	Category	Counts	Percentage of Total
Gender	Female	73	43%
	Male	95	57%
Educational attainment	University degree	126	75%
	Secondary education	42	25%
Company size	Small enterprise (fewer than 50 employees)	45	27%
	Medium-sized enterprise (51–250 employees)	29	17%
	Large enterprise (more than 250 employees)	94	56%
Organization type	Private	133	79%
	Public	35	21%

Table S2. Standardized Factor Loadings from Confirmatory Factor Analysis (CFA).

Factor	Item	Standardized Estimate	Standard Error	P-Value
Employee-Centered Artificial Intelligence Implementation (ECAII)	Item01	0.775	0.055	< .001
	Item02	0.865	0.039	< .001
	Item03	0.880	0.031	< .001
	Item04	0.889	0.027	< .001
	Item05	0.813	0.056	< .001
	Item06	0.753	0.058	< .001
	Item07	0.771	0.044	< .001
Personal attitudes toward AI	Item01	0.762	0.055	< .001
	Item02	0.731	0.051	< .001
	Item03	0.850	0.036	< .001

	Item04	0.899	0.027	< .001
Work meaningfulness	Item01	0.934	0.018	< .001
	Item02	0.889	0.031	< .001
	Item03	0.949	0.017	< .001
Job satisfaction	Item01	0.839	0.047	< .001
	Item02	0.530	0.089	< .001
	Item03	0.734	0.067	< .001
Job performance	Item01	0.631	0.086	< .001
	Item02	0.790	0.046	< .001
	Item03	0.870	0.058	< .001
